# Apex scavengers from different European populations converge at threatened savannah landscapes

**DOI:** 10.1038/s41598-022-06436-9

**Published:** 2022-02-15

**Authors:** A. Delgado-González, A. Cortés-Avizanda, D. Serrano, E. Arrondo, O. Duriez, A. Margalida, M. Carrete, P. Oliva-Vidal, E. Sourp, Z. Morales-Reyes, I. García-Barón, M. de la Riva, J. A. Sánchez-Zapata, J. A. Donázar

**Affiliations:** 1 Department of Conservation Biology, Estación Biológica de Doñana (CSIC), C/. Américo Vespucio 26, 41092 Seville, Spain; 2 Department of Plant Biology and Ecology, Faculty of Biology, University of Seville, Avda. Reina Mercedes S/N, 41012 Seville, Spain; 3 CEFE, University of Montpellier, CNRS, EPHE, IRD, Montpellier, France; 4 Instituto de Investigación en Recursos Cinegéticos (CSIC-UCLM-JCCM), 13005 Ciudad Real, Spain; 5 Department of Physical, Chemical and Natural Systems, University Pablo de Olavide, Ctra. de Utrera km. 1, 41013 Seville, Spain; 6 Department of Animal Science, Faculty of Life Sciences and Engineering, University of Lleida, Lleida, Spain; 7 Parc National Des Pyrénées, 2 rue du IV Septembre, 65007 Tarbes, France; 8 Department of Applied Biology, Miguel Hernández University of Elche, Av. de La Universidad S/N, 03202 Elche, Spain; 9 Centro de Investigación e Innovación Agroalimentaria y Agroambiental (CIAGRO-UMH), Miguel Hernández University of Elche, Elche, Spain; 10 AZTI, Marine Research, Basque Research and Technology Alliance (BRTA), Herrera Kaia Portualdea z/g, Pasaia, Spain

**Keywords:** Ecology, Agroecology, Animal migration, Behavioural ecology, Biodiversity, Biogeography, Community ecology, Conservation biology, Ecological modelling, Ecological networks, Ecosystem ecology, Ecosystem services, Grassland ecology, Restoration ecology, Ecology, Agroecology, Animal migration, Behavioural ecology, Biodiversity, Biogeography, Community ecology, Conservation biology, Ecological modelling, Ecological networks, Ecosystem ecology, Ecosystem services, Grassland ecology, Restoration ecology, Environmental sciences, Environmental impact

## Abstract

Over millennia, human intervention has transformed European habitats mainly through extensive livestock grazing. “Dehesas/Montados” are an Iberian savannah-like ecosystem dominated by oak-trees, bushes and grass species that are subject to agricultural and extensive livestock uses. They are a good example of how large-scale, low intensive transformations can maintain high biodiversity levels as well as socio-economic and cultural values. However, the role that these human-modified habitats can play for individuals or species living beyond their borders is unknown. Here, using a dataset of 106 adult GPS-tagged Eurasian griffon vultures (*Gyps fulvus*) monitored over seven years, we show how individuals breeding in western European populations from Northern, Central, and Southern Spain, and Southern France made long-range forays (LRFs) of up to 800 km to converge in the threatened Iberian “dehesas” to forage. There, extensive livestock and wild ungulates provide large amounts of carcasses, which are available to scavengers from traditional exploitations and rewilding processes. Our results highlight that maintaining Iberian “dehesas” is critical not only for local biodiversity but also for long-term conservation and the ecosystem services provided by avian scavengers across the continent.

## Introduction

Savannah ecosystems extend across tropical and subtropical regions of the world. Continuous herbaceous layers, usually dominated by grasses or sedges, and a discontinuous layer of trees and/or shrubs, as well as a deep seasonality, characterize these ecosystems and their functioning^1,2^. Humans have traditionally intervened in savannah ecosystems largely through extensive livestock grazing^3^, but the exploitation of resources by domestic herds is also regulated by seasonal changes in primary productivity. Thus, cattle carry out transhumant movements (i.e., seasonal movement of livestock usually from high mountains in summer to lowlands in winter) to access seasonal resources^4^. Despite these interventions, many savannah systems have historically maintained high levels of biodiversity as well as complex ecosystem functions^5^. However, under current scenarios of global change and modernization of agro-silvopastoral practices, the coexistence of domestic and wild ungulate populations raises important challenges for the conservation of these systems and the maintenance of their large-scale ecological processes. In particular, it is essential to determine how large vertebrates with high movement capacity respond to the spatial distribution of savannahs and to the co-occurrence of wild and domestic ungulates. It is well known that in well-structured ecosystems, where functions and interactions have not been decisively altered by humans, organisms belonging to different trophic positions, such as primary consumers, predators and scavengers, may perform long-range forays (thereafter “LRFs”)^6^ regulated by environmental changes in the availability of resources^7–9^. Thus, a large-scale approach is required to understand the importance that these LRFs might have in the ecology of the species and their long-term conservation.

Mediterranean savannahs are mid-latitude systems found in regions with mild, rainy winters and hot, dry summers^10^. They have been impacted historically by human activities through fire and grazing^11^. In Europe, Mediterranean savannahs are specifically called “dehesa/montado” (thereafter “dehesa” and *Dehesa* when as variable), which describes an agro-silvopastoral landscape today covering almost 6 million ha (10% of the total surface) in the south-western regions of the Iberian Peninsula^12^. “Dehesas” are dominated by scattered *Quercus* trees with diverse traditional uses such as livestock grazing, forestry, cork-harvesting, cereal production, and game hunting^5^. In fact, the intimate mixtures of forest and open habitat types at several spatial scales promotes landscape heterogeneity and the maintenance of an extraordinary biodiversity and well-structured food webs^13,14^. Mediterranean “dehesas” are threatened by climate change and the abandonment of traditional uses, and thus are protected under the European Habitats Directive, the basis of Europe’s nature conservation policy^13–15^. They are also defined as high nature value (HNV) farmlands where the longstanding coevolution between human societies and the environment has shaped a unique cultural landscape^15,16^.

Obligate avian scavengers (vultures and condors) are a functional group of birds that have become extraordinarily rare in recent decades around the world, with many populations virtually extinct or in serious decline^17,18^, mainly because of unnatural mortality (poisoning, electrocution and collision with powering infrastructures) and changes in traditional farming practices and human-wildlife conflicts^19–22^. This decline has repercussions far beyond the guild itself since it compromises ecosystem functions and services, such as the removal of carcasses of wild animals and livestock, which helps to prevent the spread of diseases and parasites to both wildlife and humans^23^. This process of disappearance is occurring without a precise understanding of the ecological strategies of populations of top scavengers, in particular the dynamics of space use. This is particularly relevant since large scavengers display movement strategies that involve not only local displacements, but also frequent LRFs that lead them to exploit large areas. Thus, the ecosystem functions in which they are involved and the services they provide probably develop at spatial scales of unknown range^6,24,25^. In fact, it has been postulated that the most specialist group of species (*Gyps* spp.) evolved in a context of resources (wild ungulate herds) that were highly variable in space and time, favoring life strategies based on the ability to travel long distances^7^. For example, Ruppell’s vultures *Gyps rueppellii* time their reproduction and their movements according to those of the blue wildebeest *Connochaetes taurinus* and other large migratory mammals^26–28^.

In this context, it is essential to examine whether the existence of “dehesas” in southern Europe shapes movement patterns of large body-sized avian scavengers on a large geographic scale. Former approaches to understanding the role of the Iberian “dehesas” in the use of landscapes by avian scavengers relied merely on a small-scale approach^29,30^, but their importance could be much greater if “dehesas” govern LRFs from distant colonies and breeding areas. If so, this would reveal a new dimension in the understanding of the ecological and functional role of this globally threatened group of vertebrates. This is particularly relevant given that currently the conservation of vultures lies almost exclusively on the protection of breeding sites and the maintenance of supplementary feeding points at local or regional scales^31–34^. Clearly, larger-scale approaches, such as the identification of areas visited by individuals from different populations, are necessary. Similar approaches have been taken for seabirds in marine environments^35^ and can help focus transboundary conservation actions^36^.

With these aims, we took advantage of unprecedented information on the movements of 106 GPS-tagged adult Eurasian griffon vultures (*Gyps fulvus*) belonging to five populations located in the Iberian Peninsula and southern France, to assess the importance of the “dehesas” in their foraging movements. We chose the Eurasian griffon vulture as a target species due to its main role as a provider of key ecosystem services within Mediterranean landscapes^37–39^. We focus on how the maintenance of traditional habitats and agro-silvopastoral uses of “dehesas” attract vultures from distant populations, identifying how individual traits and social and environmental factors govern vultures' movements. Our results highlight the crucial importance of the Mediterranean “dehesas” for top-scavenger populations of Western Europe and call for the necessity to design novel policies considering the socio-ecological importance of these singular systems.

## Results

**Figure 1:**
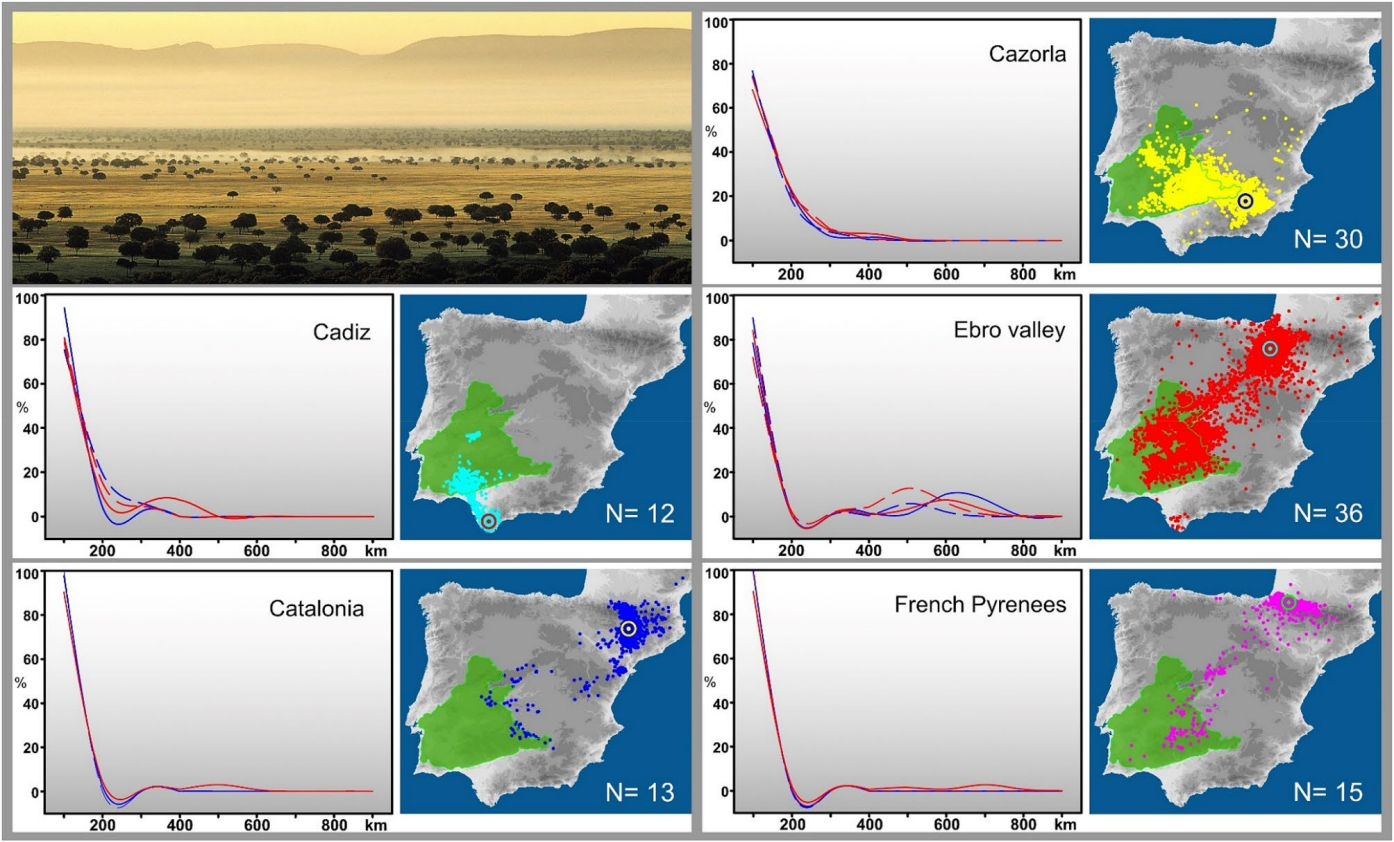
Study populations and areas. Above left: “dehesa” landscape in central Iberia (Cabañeros National park) (Photo: V. García-Canseco). We show a panel for each study population with both a map of the daily locations and the splined distribution of their distances to the nest or activity center. Continuous and discontinuous lines represent males and females respectively (Winter in blue, summer in red). The mean position of nests/activity centers for each population is shown by a circle. The distribution of the “dehesas” in the Iberian Peninsula is shown in green (depicted from CORINE land Cover). Maps were generated using QGIS 3.6.0 Noosa (https://www.qgis.org/es/site/)^90^.

**Figure 2:**
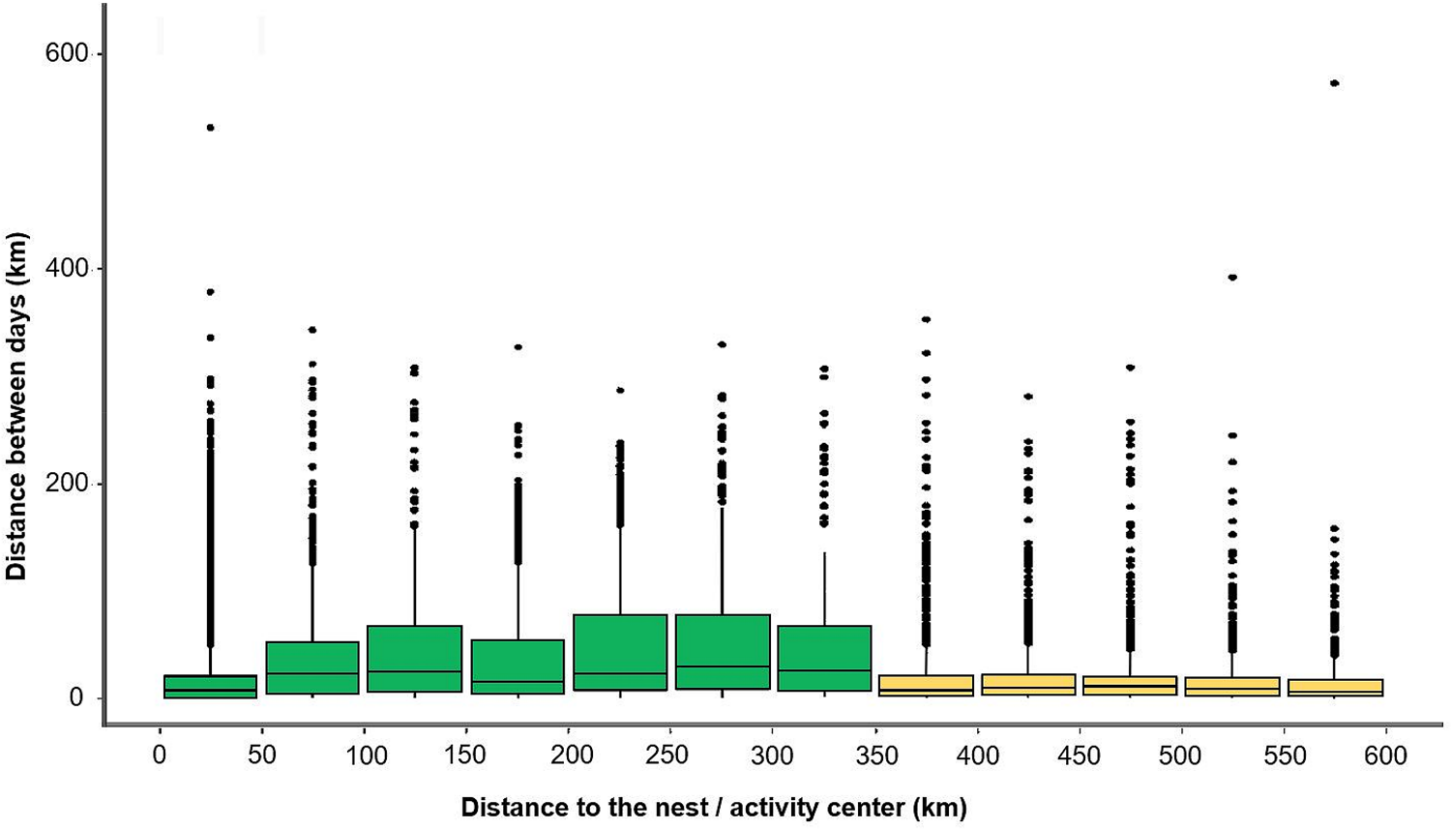
Daily distance travelled by GPS-tagged Eurasian griffon vultures. We show Euclidean distances (in km) between locations of the GPS-tagged birds (all pooled) in consecutive days (from day_t_ to day_t+1_) in relation to the distance from the location of the day_t+1_ to the nest or activity center. Colors show categories (< 350 km or > 350 km) used in the modelling procedures.

Although most daily locations (92.1%, N = 58,941) were < 350 km from the central place (nest/activity center) of the GPS-tagged vultures, 32 out of the 106 birds belonging to the five populations moved beyond this limit, as far as 800 km, all directed to southwestern Iberia, a region dominated by “dehesas” (Fig. 1). In fact, short-range displacements between consecutive days occurred at both nearby (<50 km) and distant (>350 km) locations from the central place, so that intermediate locations were interpreted as travel journeys from breeding to distant foraging areas. In other words, these were days in which birds prioritized directional movements over foraging, to reach those areas >350 km (Fig. 2). Both males and females spent more days in summer than in winter beyond the limit of 350 km (see details in Supplementary Table 2).

The first GLM procedure showed that the proportion of days that the individual was more than 350 km away from its central place was related to *Season* and *Sex* and their interactions (Supplementary Tables 4-5), showing that LRFs were carried out more often by females and during the summer. Noticeably, the variable *Population* was non-informative, suggesting that the detected pattern was similar for individuals from all the study areas. The analysis that focused on the Ebro valley population (the population with the most GPS-tagged Eurasian griffon vultures (N = 36) and the largest number of individuals (N = 13) that made LRFs, i.e., >350 km (Table 1 and Supplementary Table 3)) showed that females and non-breeding individuals (52.8% of the total) performed these LRFs more frequently (Supplementary Tables 6-7). In fact, four breeding females with growing chicks made LRFs (> 350 km) to southwestern Iberia during the rearing period (Supplementary Fig. 2).

**Table 1:** Locations, sample sizes and characteristics of the different study populations. Central position of the study populations; tracking periods, number of Eurasian griffon vultures tracked and number of GPS positions analyzed (one position/day/individual). All the individuals were resident adults.

Study area	Latitude	Longitude	Tracking period	N of Eurasian griffon vultures	GPS positions
Ebro Valley	42.263	−1.572	2015–2018	36	25,950^a^
Cádiz	36.323	−5.711	2018	12	2,998^b^
Cazorla	37.960	−2.936	2014–2019	30	22,894^a^
Catalonia	42.367	0.705	2018	13	2,812^c^
French Pyrenees	43.070	−0.415	2013–2018	15	4,332^d^

^a^Solar-powered GPS/GPRS-GSM (e-obs) (90 g). https://e-obs.de/.

^b^Solar-powered GPS-GSM Backpack series Griffon LF (Ecotone) (34 g). http://ecotone-telemetry.com/en.

^c^Solar-powered GPS/GMS OrniTrack-50 (Ornitela) (50 g). https://www.ornitela.com/.

^d^Solar-powered GPS-zigbee loggers (UvA-BiTS) (55 g)^91^. https://www.uva-bits.nl/.

**Figure 3:**
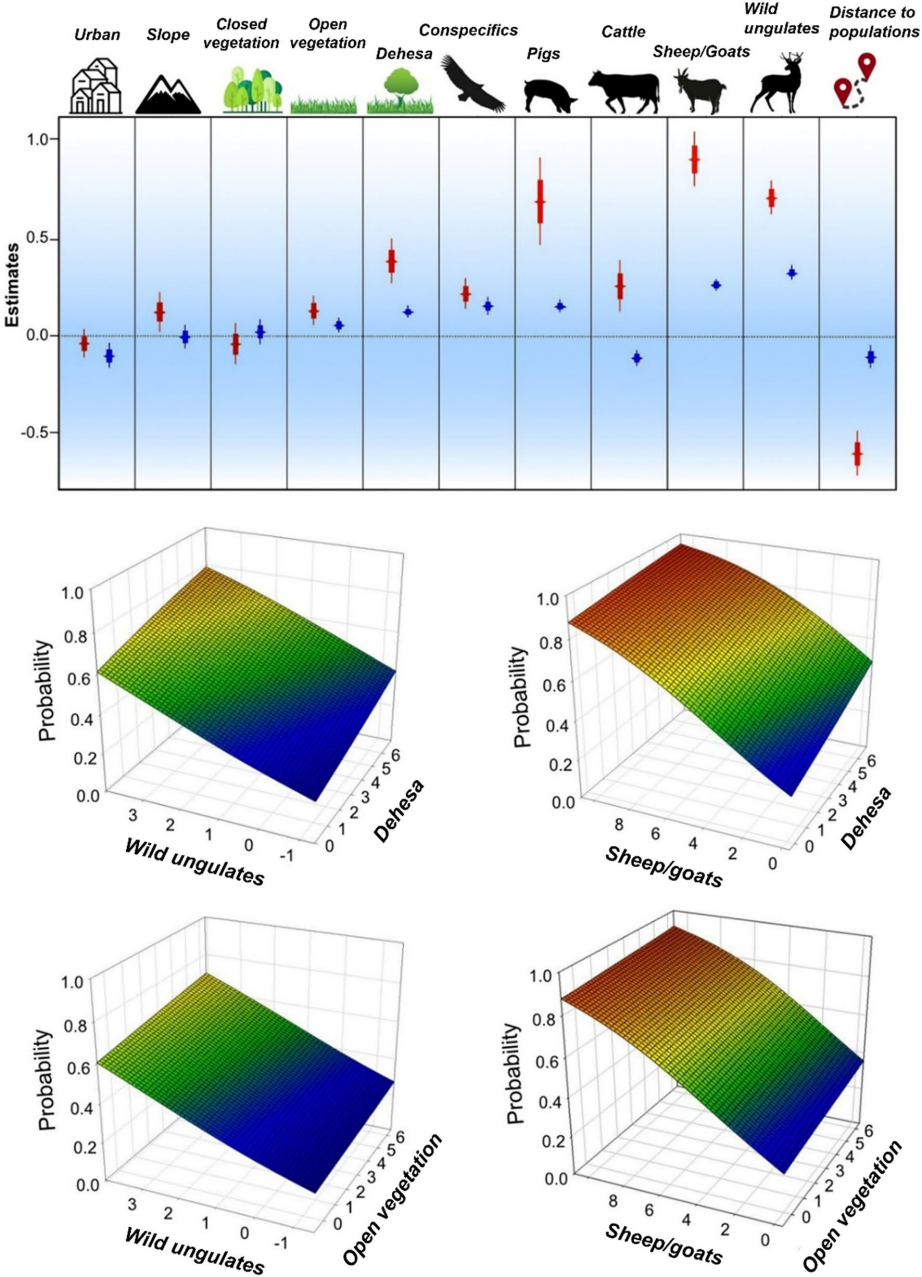
Coefficient plot for models evaluating spatial coincidence between populations. Thick and narrow bars represent respectively standard errors and confidence intervals (95%) of the estimates of variables within models evaluating the probability of presence of GPS-tagged Eurasian griffon vultures in a 10 × 10 km grid cell covering peninsular Spain (red) and the coincidence of vultures from different populations in the same grid cell (blue). Below we show the projection of the probability of coincidence in relation to the most relevant variables describing habitat (*Dehesa* and *Open vegetation*) and food resources (*Sheep/goats* and *Wild ungulates*).

The second group of analyses corresponded to the fitting of two GLM models for (i) the *Probability of presence* of at least one GPS-tagged Eurasian griffon vulture and (ii) the *Number of populations* of origin of the individuals that had visited each 10×10 grid cell. In both cases, the probability was higher when there was a greater percentage of *Open vegetation* and *Dehesa*, larger numbers of *Wild ungulates* and livestock (i.e., *Sheep/goats, Pigs, Cattle*), closer to the monitored Eurasian griffon vulture populations and with a higher potential density of foraging vultures (i.e., *Conspecifics*) estimated from national censuses of the breeding populations of the species (Supplementary Tables 8-9). In contrast, the opposite effect of *Cattle* positively determined the *Probability of presence* of Eurasian griffon vultures but negatively determined the *Number of populations* sharing the same area. It should also be noted that ungulate abundance and habitat showed an additive effect although with different effect sizes. Finally, the *Probability of presence* of Eurasian griffon vultures was greater in rough terrain (i.e., larger values of *Slope*) with *Open vegetation*, while the overlap of individuals belonging to different populations (i.e., *Number of populations*) was higher in less urbanized areas (i.e., lower values of *Urban*) (Fig. 3 and Supplementary Tables 10-11).

## Discussion

Our study shows that foraging Eurasian griffon vultures from five populations located across Western Europe made LRFs to converge in the same region of the Iberian Peninsula. This pattern was common to all the populations and, because it was shown by adult resident birds, cannot be explained by migratory or dispersal-prospective movements described in other scavenger species^6,40^, but occurs only during the pre-adult stage in our target species^41^. We also show that the existence of “dehesas” together with the availability of food resources were the main factors triggering the selection of these common foraging grounds. Our findings may constitute a key piece that connect the conservation of the “dehesa”*,* a high value farming system that requires high priority in conservation^42^ with the maintenance on a continental scale of populations of endangered large avian vertebrates and the ecosystem services that they supply. Our results emphasized the role of two types of variables (*Wild ungulates* and livestock (i.e., *Sheep/goats*, *Pigs*, and *Cattle*) abundance, and *Dehesa*). We hypothesized therefore that the joint effect of the simultaneous presence of both factors implied an environmental incidence greater than the sum effect of factors considered in isolation. This is likely because the variable *Dehesa* accounted for environmental factors not evaluated in our analysis, which would determine an added value improving the potential quality of the patches for the Eurasian griffon vultures. In this traditional Mediterranean agro-silvopastoral system, the livestock species complement each other; whereas ruminants use pastures, harvested cereal crops, stubble, and fallow land, the Iberian pigs (*Sus scrofa domestica*) consume *Quercus* acorns. This complementarity probably determined a greater availability of resources, more complex than that measured by our biomass index, due to the different mortality patterns of the ungulate groups^43^. For example, the abundance of cattle, the species that contributes to the bulk of ungulate biomass, was nevertheless the variable that has the smallest effect size on the *Presence of vultures*. Surprisingly, it had also a negative effect on the *Number of populations* visiting the grid. The effect on presence is likely to be small because cattle carcasses, unlike other livestock species, are often disposed and hence rarely available to vultures^44^, so perhaps they are only exploited by birds from nearby populations. The same reasoning could explain why vultures from few populations visited the cells with an abundance of cattle.

Moreover, many “dehesas” are rich in wild ungulates^13^, whose natural mortality and big game hunting provides scavengers with important trophic resources^28,45^. Wild herbivores, in addition, are rapidly expanding due to rewilding processes of Mediterranean habitats so that currently south-western Iberian regions maintain a rich community of large body-sized primary consumers^46^. In this context, the management of carcasses from livestock and game hunting in the “dehesas”, and other Mediterranean woodlands, can also be determinant in our findings. Due to limited access to mechanized collecting systems, livestock carcasses, except for cattle, are usually available to scavengers, which has also been reinforced by recent more permissive sanitary regulations^44,47^. Apart from this, avian scavengers may also consume game remains, which, after the hunts, are usually moved to supplementary feeding sites within hunting properties.

As a whole, and as has been proposed for other systems^48,49^, the complementarity of resources provided by wild and domestic ungulates may be especially profitable for avian scavengers, as it would buffer environmental fluctuations that would be spatially or temporally limiting. Finally, it is important to point out that the system is dynamic over time and that it will probably be subject to important changes in the medium- and long-term due to the abandonment of traditional agro-silvopastoral systems and generalized rewilding processes^50^. In this context, it is foreseeable that in the future the relative importance of wild ungulates and of hunting activities in the foraging strategies of avian scavengers of Mediterranean ecosystems will increase with potential consequences on populations and guilds that will need to be considered^30,51,52^.

Most of the LRFs recorded during our study occurred during warm months, when updrafts from warm air make flight conditions for vultures more favorable^53^. As we expected, more non-breeding Eurasian griffon vultures made these kinds of movements as they were not attached to reproductive tasks. Very interestingly, these movements involved more females than males, something very evident in the data from the Ebro valley population, with a larger sample size and a more equal balance between sexes. Other authors also found that these movements were more frequent among females arguing that mate searching may be motivating the observed patterns^6^. In our case, however, as we pointed out above, this hypothesis does not seem plausible because all the GPS-tagged Eurasian griffon vultures were adults breeding nearly regularly or had their activity centers in their populations of origin. Even more interesting is the fact that active breeding females, contrary to males, also performed short-duration travels to these distant foraging zones during the chick-rearing period. It can be hypothesized that there could be a sex-asymmetry in the foraging strategy similar to that observed in large seabirds^54^. Both, seabirds and vultures, forage over large areas searching for ephemeral clumped food resources, and so there may be parallel strategies in the exploitation of space at a large scale. In this context, and similarly to seabirds^55^, vultures may use a double strategy mixing short and long foraging trips, the latter enabling adult birds to refuel their body reserves on high-quality patches, especially if they are depleted because of the high requirements of breeding tasks^26^. From a conservation point of view, sex asymmetries in habitat use at any scale can determine different risks for both sexes from anthropogenic changes^56^ with potential consequences in population viability^57^.

The association between the density of conspecifics and the probability of use of common areas should also be highlighted. This finding could indicate that social aspects play an important role in the exploitation of resources. On the one hand, it is well known that *Gyps* vultures rely on social information to locate carcasses, even more so when they are randomly distributed in space, as is the case of extensive livestock and wild ungulates^52,58–60^. It is also increasingly evident that the social life of vultures goes far beyond trophic aspects, playing an essential role in numerous aspects of life strategies like breeding and mating systems, foraging techniques, social hierarchies, gathering behaviour, and interspecific interactions^61^.

Finally, we should consider our findings within an eco-evolutionary perspective in which seasonal migratory movements of ungulates would have triggered parallel strategies in avian scavengers. In fact, our findings mimic similar large-scale movements to those of large body-sized avian scavengers in other biomes in pursuit of herds of herbivores that move seasonally in search of grasses^7^, but see^28,62,63^, and far outweigh the previously short-distance movements of vultures following transhumant livestock reported in regions of Iberia^64,65^. This raises the question of whether the LRFs of top scavengers have a common ecological basis on both continents. Indeed, in our study area, for millennia, and until very recent times, millions of livestock from summer mountain areas made seasonal movements to winter in the Iberian south-western “dehesas”^66^. We can therefore consider whether LRFs of the Eurasian griffon vultures from regions of southwestern Europe and their convergence in the “dehesas” system could respond to evolutionarily fixed behaviors in a scenario as was above described.

### Perspectives

Our findings show the importance of Mediterranean “dehesas” for the management and conservation of vulture populations throughout their distribution area across Western Europe. This information must be incorporated into European vulture conservation strategies, which currently focus primarily on the protection of breeding sites and on the provision of safe food resources via supplementary feeding points^31,32^. These latter measures, moreover, are applied close to the breeding colonies, without considering that foraging vultures move through much broader areas where they are subjected to a multiplicity of risks^67^. In addition, and focusing on a debated topic^68^, one might wonder if the coincidence of vultures from very distant breeding areas in the Iberian savannahs would promote social and demographic connectivity between populations of Western Europe, which in the end, could lead to an improvement in its viability.

In light of our results, we propose that the delineation and monitoring of the Iberian “dehesas” should be a top priority in developing conservation strategies for avian scavengers, as has been projected for other organisms exploiting resources in large areas ^69,70^. In this respect, it is important to remark that the Mediterranean “dehesas” located in the Iberian Peninsula hold a very important fraction of the European populations of other endangered avian scavengers such as cinereous vultures (*Aegypius monachus*) as well as endemic globally endangered top predators such as Spanish imperial eagles (*Aquila adalberti*) and Iberian lynxes (*Lynx pardinus*)^13,29^.

From an ecosystem perspective, the management of “dehesas” still requires much improvement as they are threatened by overexploitation, mishandling, and replacement by intensive livestock farming practices^71^. As occurs with other high natural value farmlands, this compromises the provision of ecosystem services and the maintenance of their socio-ecological viability^16^. “Dehesas” and, in general, savannah systems, are examples of the coexistence of biodiversity and sustainable human economies favoring the maintenance of large spatial-scale processes such as top-scavenger movements, which may be key to maintaining functions and services within ecosystems^42^. Future conservation strategies for Iberian “dehesas” must consider the existence of organisms moving and supplying services on a large scale, so any approach must be transboundary between regions and countries, operating outside of local administrative constraints.

## Materials and methods

### Experimental design

#### Data set

We marked 106 Eurasian griffon vultures in five regions of the Iberian Peninsula and southern France (Table 1, Fig. 1). We consider them as representative of the variability found in the original distribution area of the species in Western Europe as they cover almost the entire range occupied. They are also among the most important from a numeric point of view^72^ (Fig. 1). In all populations, we focused on adult birds (>5 yr. old) that were identified by plumage features^73^. All the GPS-tagged Eurasian griffon vultures were considered as residents as they were recorded attempting to breed within a 50 km radius from the trapping points in years after marking. In those years, when they did not nest, the individuals maintained their main activity centers in colonies also within this same area^56,67,74^). The solar-powered devices weighed between 30–100 g and were attached using back-pack harnesses with a total weight of the system between 1–2.5% of the total weight of the bird. Tracking periods were equal to or greater than one year (Table 1). Device configuration was dependent on season and day length, providing locations every 5–10 min, more frequently during the warmest and sunniest months^75^.

#### Tracking data

For each tracking day, we selected a single location per day and individual, namely the GPS position closest to solar noon within the 10.00–14.00 h interval, corresponding to the hours of maximum flight activity. Due to the well-known existence of strong seasonal patterns in the individual movements of Eurasian griffon vultures^24,30^ we classified the locations into two differentiated seasons, namely: summer (March-September) and winter (October-February). The final data set for analyses comprised 58,986 GPS locations.

For each breeding individual, we determined the position of its central place, which could be its nest or the activity center in each year of study. Nests were located through both field observations^72^ and/or the analysis of the accelerometer sensor positions during the incubation season. As a result, we were able to establish the nest position per year for at least 74 individuals (70% of the total, N = 106). For those birds not breeding in a particular year, we calculated the annual activity center as the mean of the locations from dusk to dawn hours (resting zones).

### Statistical analysis

First, we used a Generalized Linear Mixed Model to examine differences in LRFs among individuals, populations, and between seasons. The response variable was the number of days that the individual was more than 350 km away from its central place in relation to the total number of days with locations in each season (binomial error, logit link function). This 350 km cut-off point was defined based on the distribution of distances to the central place of the daily position *vs.* the distance traveled by the individual from the previous day (Fig. 2). Consequently, this boundary represented the distance at which movements between days decline sharply, indicating that the Eurasian griffon vultures, after the displacement flights, remained in these new foraging areas. Therefore, it was a good threshold to separate directional movements for moving between areas from routine foraging movements within a specific area. Models included the following explanatory variables: (i) *Sex (Male/Female)*: Previous research on the Eurasian griffon vulture populations showed that males exploit areas more transformed by human activities (urbanization, infrastructure) apparently assuming higher mortality risks^56,67^. Combined with the fact that females generally move longer distances than males in raptors^76^, we can predict that females should make more frequent use of distant areas where human disturbance is lower, such as “dehesas”. (ii) *Season (Summer/Winter)*: Because vultures rely mainly on soaring flight, more LRFs are expected in summer, when the availability of thermal currents is higher ^74,77^. (iii) *Population (Cadiz/Cazorla/Ebro valley/Catalonia/French Pyrenees)*: In principle, no differences between populations can be predicted in the proportions of individuals performing LRFs. We also tested the effect of the interactions *Sex:Season* and *Sex:Population*. *Individual* and *Year* were included as random terms to account for the non-independence of the data.

Additionally, to delve more deeply into these results, we focused on the study population of the Ebro valley (with the largest sample size (Table 1) and complete information on individual breeding success). We performed a GLM (binomial error, logit link function) to determine if the probability of making at least one LRFs (≥ 350 km) per year was dependent on *Sex (Male/Female)* and *Breeding success (Yes/No)*. We considered that a vulture had success when a fledgling was observed in the nest. We predicted that individuals that have failed to reproduce were more likely to perform LRFs.

Second, for the whole study period, we analyzed the environmental drivers explaining the habitat use of GPS-tagged Eurasian griffon vultures using the 10×10 km grid cells from mainland Spain (UTM grid cells). We performed two analyses corresponding to two response variables: (a) the *Presence/Absence* of GPS-tagged Eurasian griffon vultures (Generalized Linear Model, binomial error distribution, and logit link function). In this case, the response variable was “1” when at least one vulture of any population visited the grid cell and “0” when no vulture of any population was recorded, and (b) the *Number of populations* of origin of these individuals. In this case, the response variable was the number of populations. It took values between 0/5 (no vultures of any populations were detected in the grid cell) and 5/5 (vultures of the five populations were detected in the grid cell). We used a binomial error (log link function). Based on previous studies^78^ and references therein, we chose a primary set of thirteen explanatory variables (see Supplementary Table 1 and Supplementary Fig. 1) describing the habitat (physiography and vegetation), the degree of human disturbance, the availability of trophic resources (domestic or wild ungulates), and the strength of intraspecific competition. As a whole, we can predict that those grid cells with more appropriate habitats for searching activities (open landscapes) and where there is availability of potential food (wild and/or domestic ungulates), will be more visited. Thus, the “dehesas”, which meet these requirements, are expected to be positively selected^29,30^. On the other hand, the probability should also be higher where human transformation of the landscape is low^79^. Finally, grid cells where the density of vultures is higher will be more visited since the Eurasian griffon vultures rely on social cues for the location of food (see above). Models also included the *Mean distance* between each grid-cell to the five study populations. Logically, it can be expected that grid cells will be less visited when more isolated (far away) from the potential origin of the birds.

As a previous step in the modeling, we assessed collinearity between explanatory variables by calculating the Spearman correlation coefficients for all pairwise combinations of predictors. Those exceeding |r| > 0.5 were considered redundant, so the least biologically meaningful variable was excluded from further analyses^80^. Comparisons between competing models were based on the small-sample-size corrected version of the Akaike information criterion (AICc). Competing models (i.e., models differing <2AICc) were averaged^81,82^ Finally, we tested for overdispersion, and we determined pseudo-R-Squared. Model fits were assessed using *DHARMa*^83^. All the analyses were done using R version 3.5.1 (https://www.r-project.org/)84 and the packages *MASS*^85^, *lme4*^86^, *gmodels*^87^, *AICcmodavg*^88^, *MuMIn*^89^. Maps were generated using QGIS 3.6.0 Noosa (https://www.qgis.org/es/site/)^90^.

### Ethical statements

All methods and procedures were performed in accordance with the relevant guidelines and regulations, followed the protocols approved by the Consejo Superior de Investigaciones Científicas (CSIC) Ethics Committee (CEBA-EBD-12-56) and were authorized by the Gobierno de Navarra (Ebro Valley), Generalitat de Catalunya (Pyrenees), Junta de Andalucía (Andalusia), and Centre de Recherches sur la Biologie des Populations d'Oiseaux (CRBPO)—CNRS/MNHN (France).

## Supplementary Information

Supplementary Information.

## Data Availability

Data underlying the study will be deposited at Digital.CSIC, the institutional repository of the Spanish National Research Council (https://www.csic.es/en/open-science/access-digitalcsic).
