# Supplementary material for: Apex scavengers from different European populations converge at threatened savannah landscapes
Source: Sci Rep. 2022 Feb 15;12:2500. doi: 10.1038/s41598-022-06436-9 (PMC8847400; doi:10.1038/s41598-022-06436-9)
Supplement: Supplementary file 1 — Supplementary Information. [file 41598_2022_6436_MOESM1_ESM.docx]

**Supplementary Material for:**

**Apex scavengers from different European populations converge at threatened savannah landscapes**

**Authors**

A. Delgado-González^1*^, A. Cortés-Avizanda^1,2^, D. Serrano^1^, E. Arrondo^1,8,9^, O. Duriez^3^, A. Margalida^4^, M. Carrete^5^, P. Oliva-Vidal^6^, E. Sourp^7^, Z. Morales-Reyes^8,9^, I. García-Barón^10^, M. de la Riva^1^, J. A. Sánchez-Zapata^8,9^, J. A. Donázar^1^

**Affiliations**

*. Corresponding author: a.delgado2096@gmail.com

1. Department of Conservation Biology, Estación Biológica de Doñana (CSIC), C/. Américo Vespucio 26, E-41092 Sevilla, Spain.

2. Department of Plant Biology and Ecology, Faculty of Biology, University of Seville, Avda. Reina Mercedes s/n, 41012, Sevilla.

3. CEFE, University of Montpellier, CNRS, EPHE, IRD, Montpellier, France.

4. Instituto de Investigación en Recursos Cinegéticos (CSIC-UCLM-JCCM), E-13005 Ciudad Real, Spain.

5. Department of Physical, Chemical and Natural Systems, University Pablo de Olavide, Ctra. de Utrera km. 1, E-41013, Sevilla, Spain.

6. Department of Animal Science, Faculty of Life Sciences and Engineering, University of Lleida, Lleida, Spain.

7. Parc National des Pyrénées, 2 rue du IV Septembre, 65007 TARBES, France.

8. Department of Applied Biology, Miguel Hernández University of Elche, Av. de la Universidad S/N, E-03202 Elche, Spain.

9. Centro de Investigación e Innovación Agroalimentaria y Agroambiental (CIAGRO-UMH), Miguel Hernández University of Elche, Elche, Spain

10. AZTI, Marine Research, Basque Research and Technology Alliance (BRTA), Herrera Kaia Portualdea z/g, Pasaia, Spain.

**This file includes:**

Supplementary Figs. 1, 2

Supplementary Tables 1 – 11

References 37, 72, 78, 89, 90, 92 – 94

**Supplementary Fig. 1**

Spatial variation in the values of both the response and the explanatory variables fitted to the GLM models aimed to evaluate the presence and co-occurrence of populations of GPS-tagged Eurasian griffon vultures in the 10 x 10 km grid covering peninsular Spain. Maps were generated using QGIS 3.6.0 Noosa (https://www.qgis.org/es/site/)^90^.


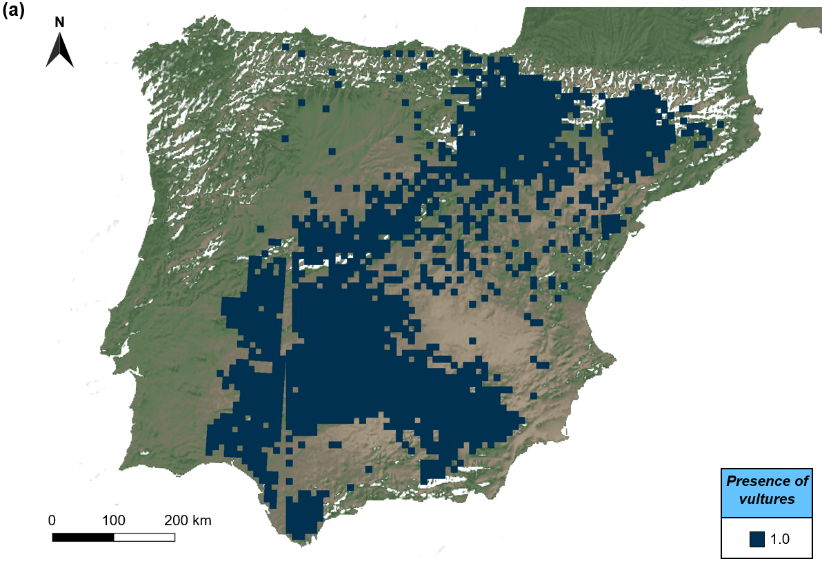

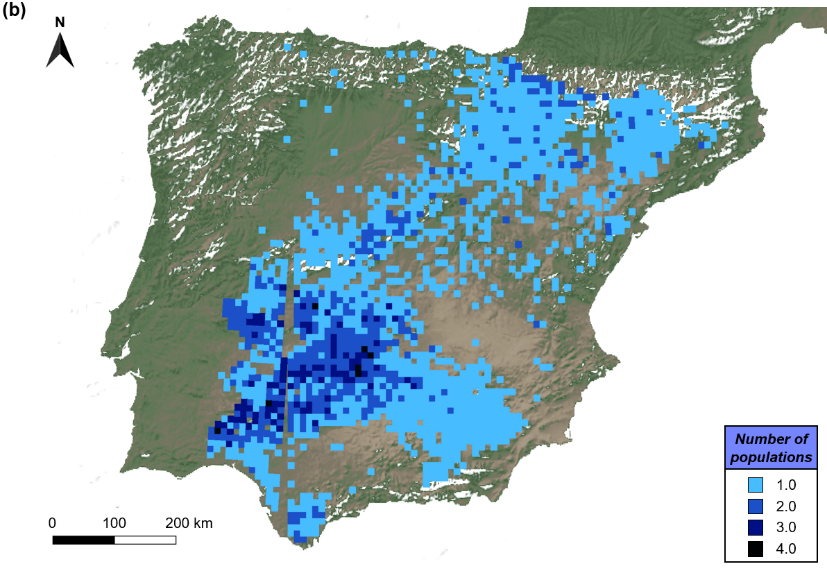

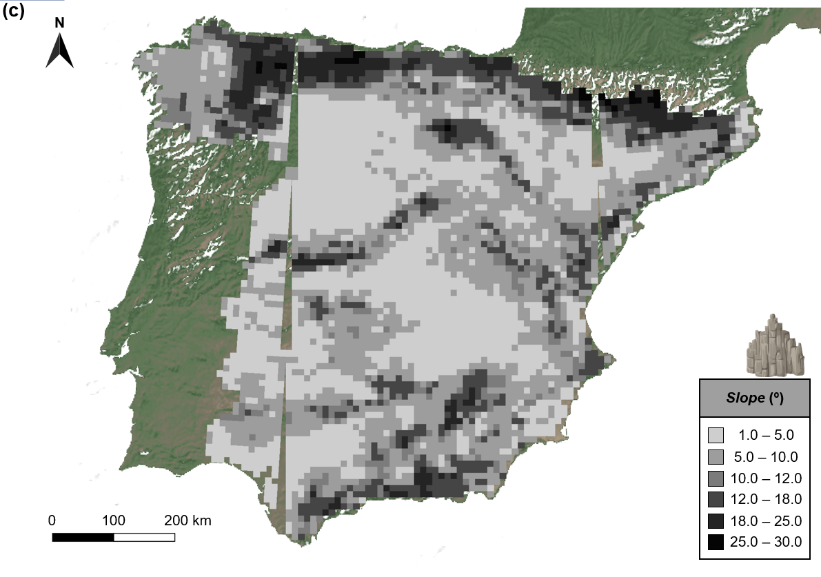

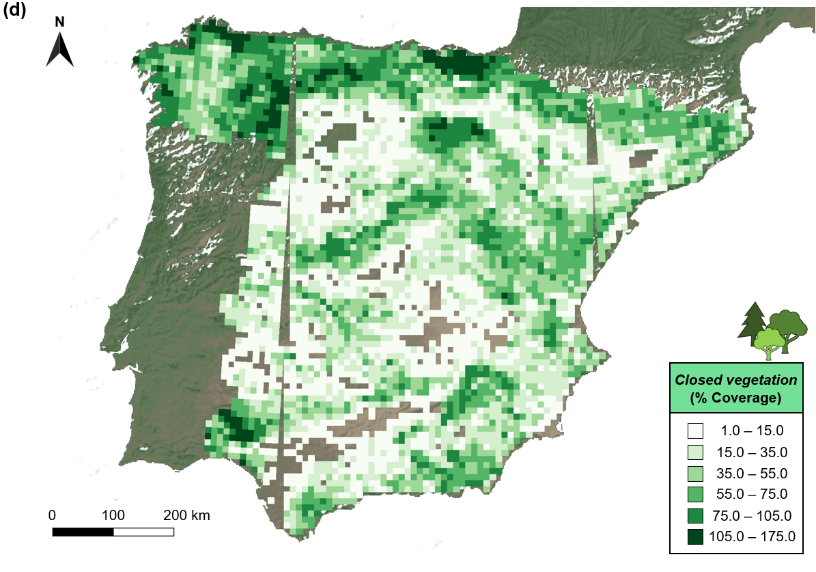

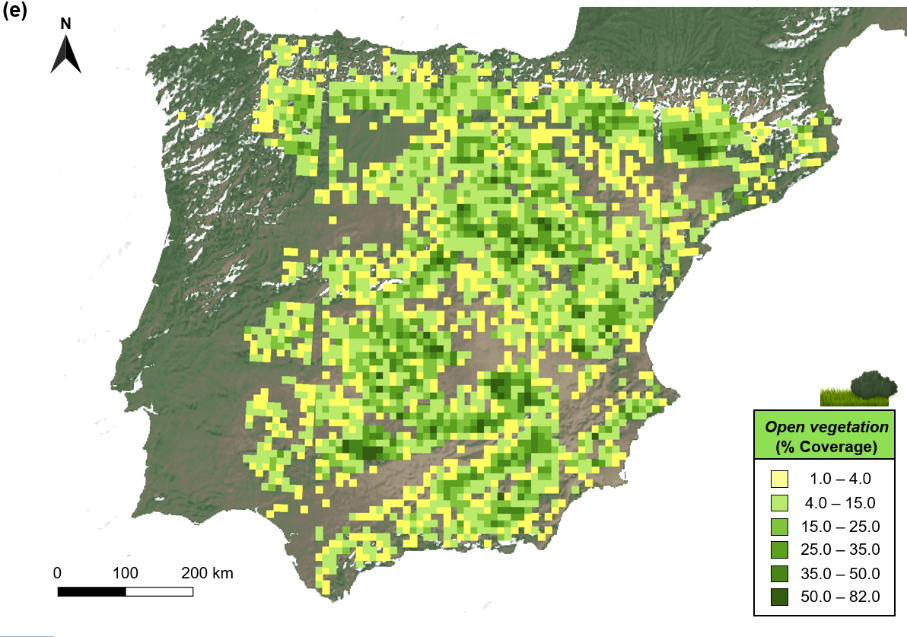

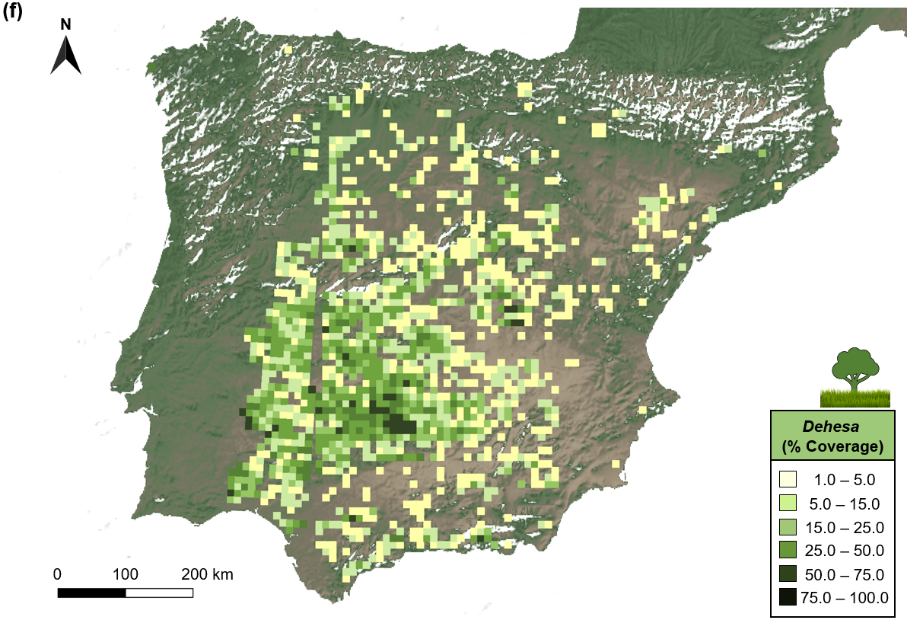

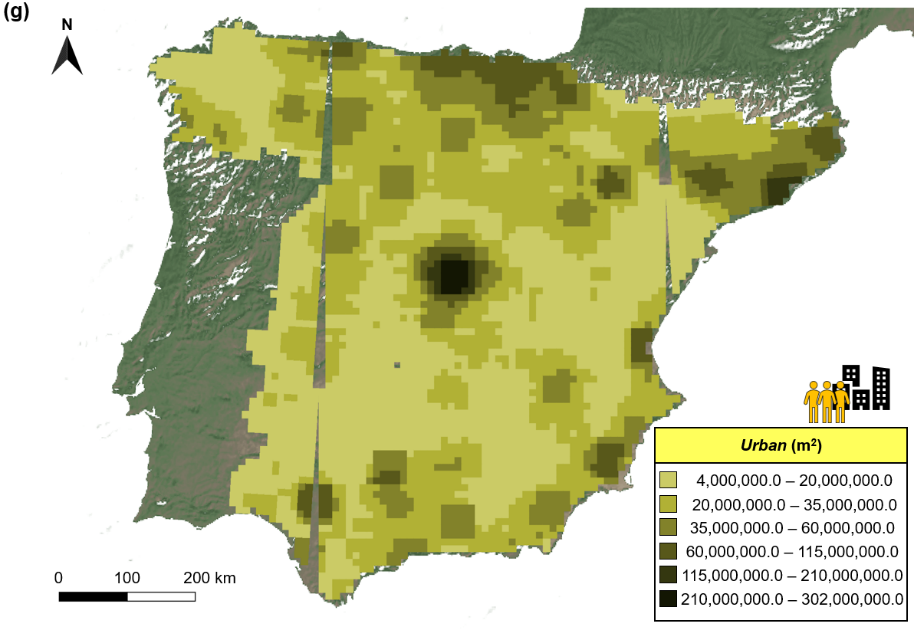

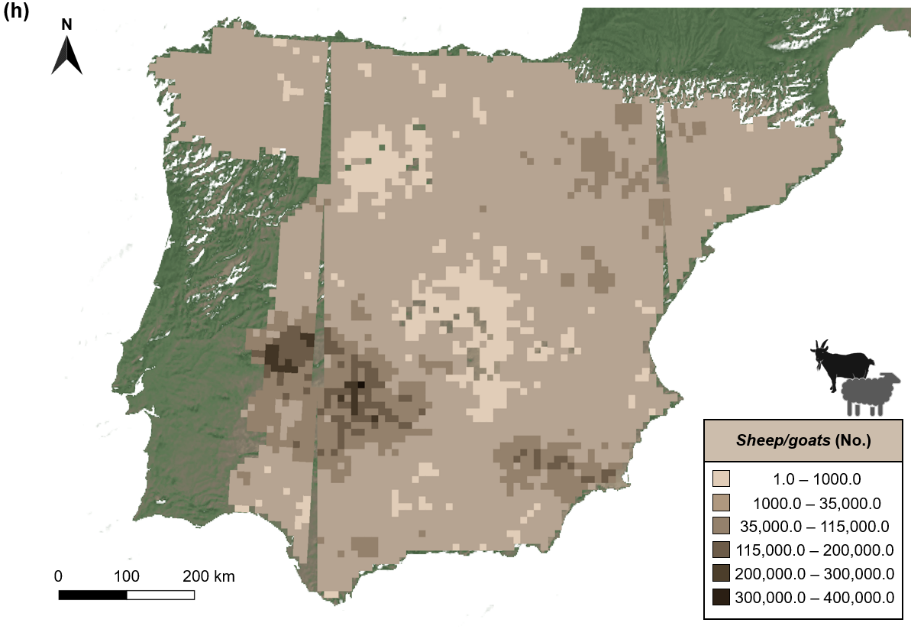

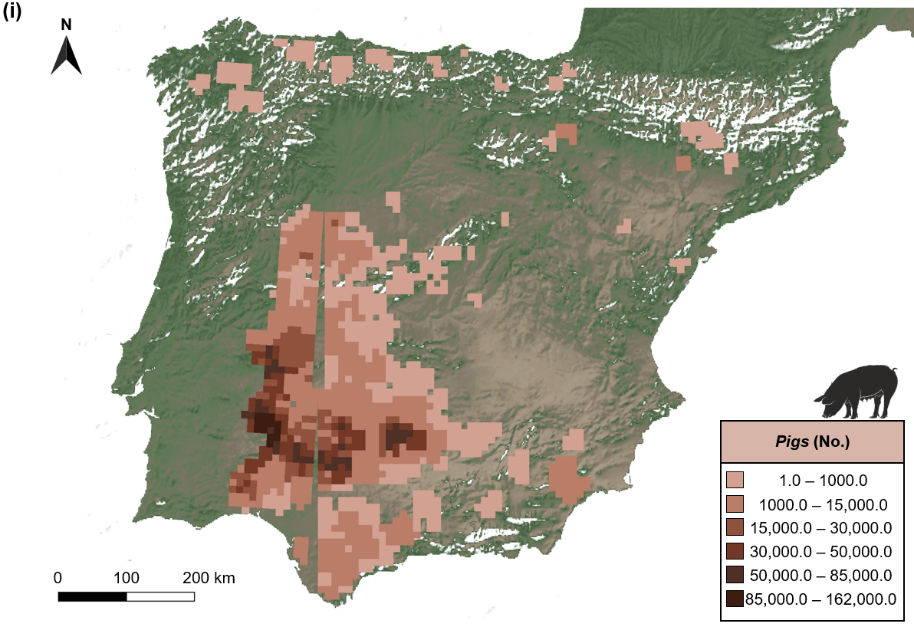

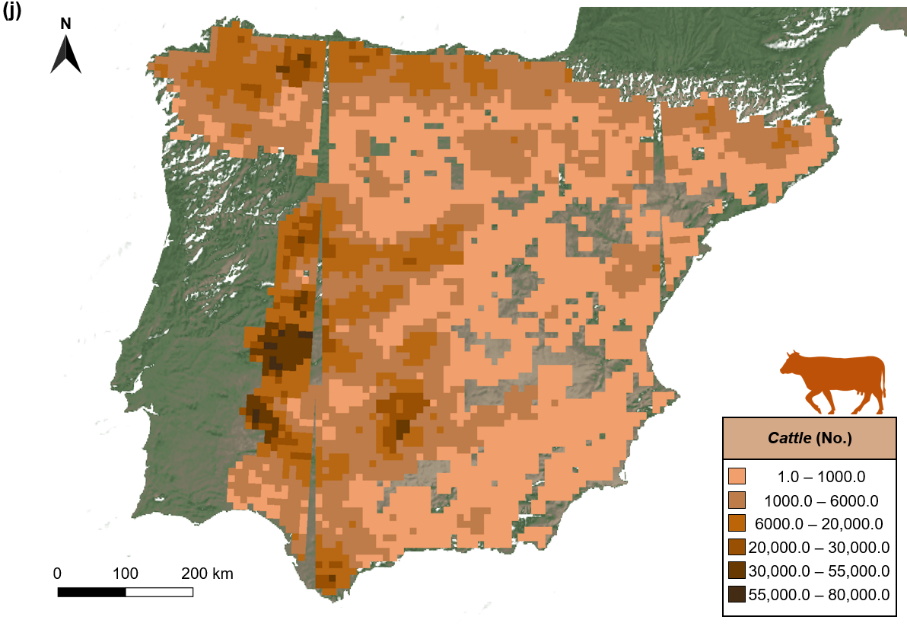

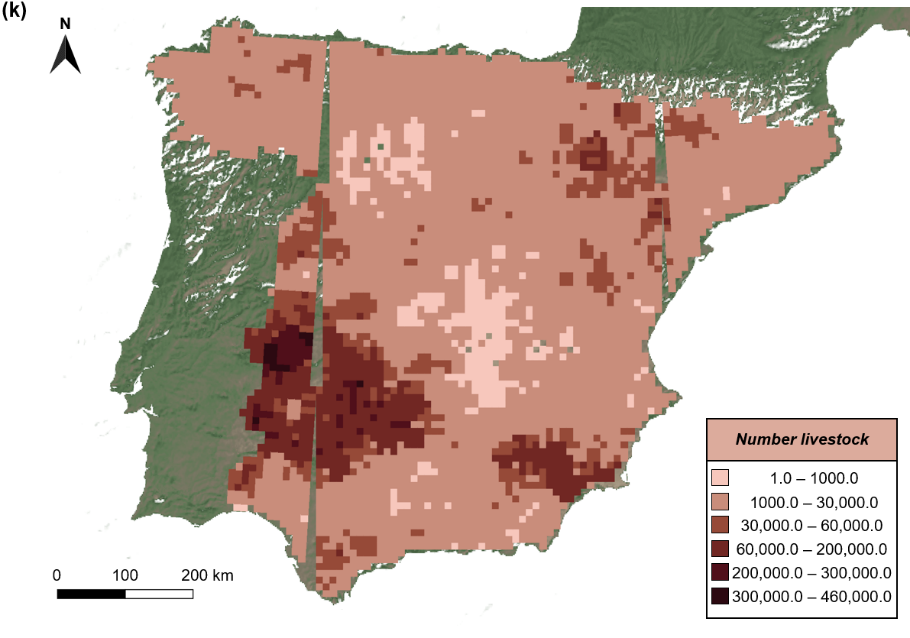

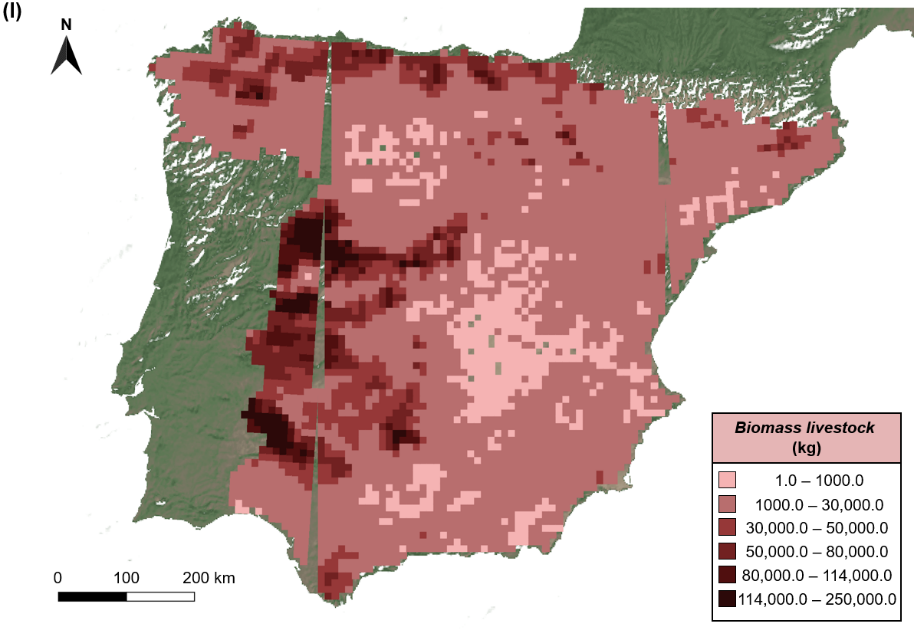

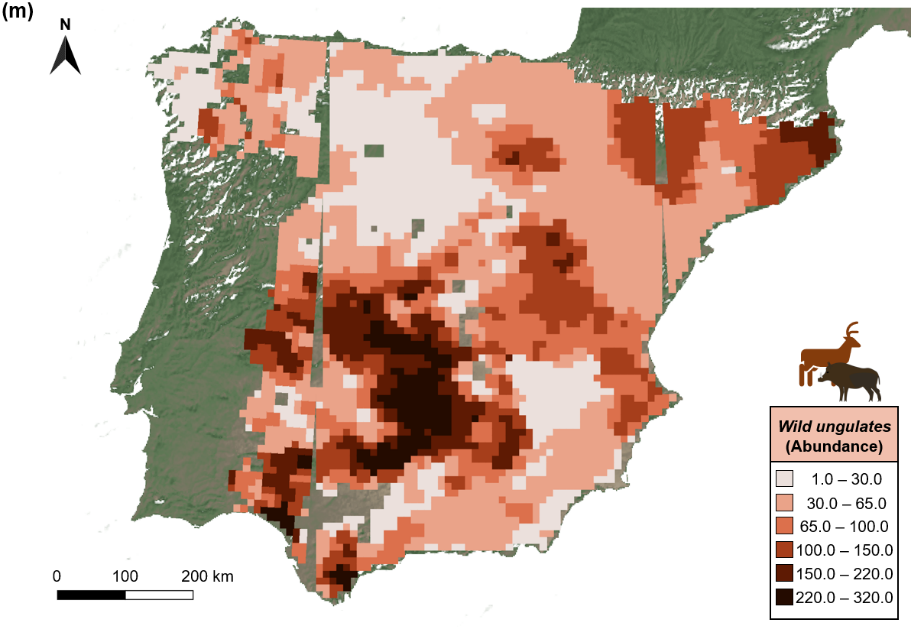

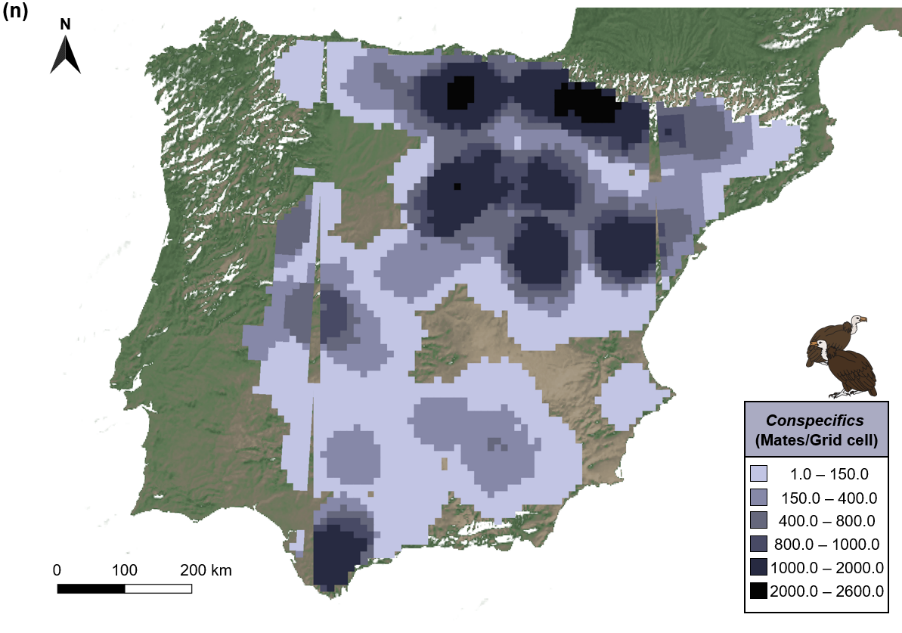


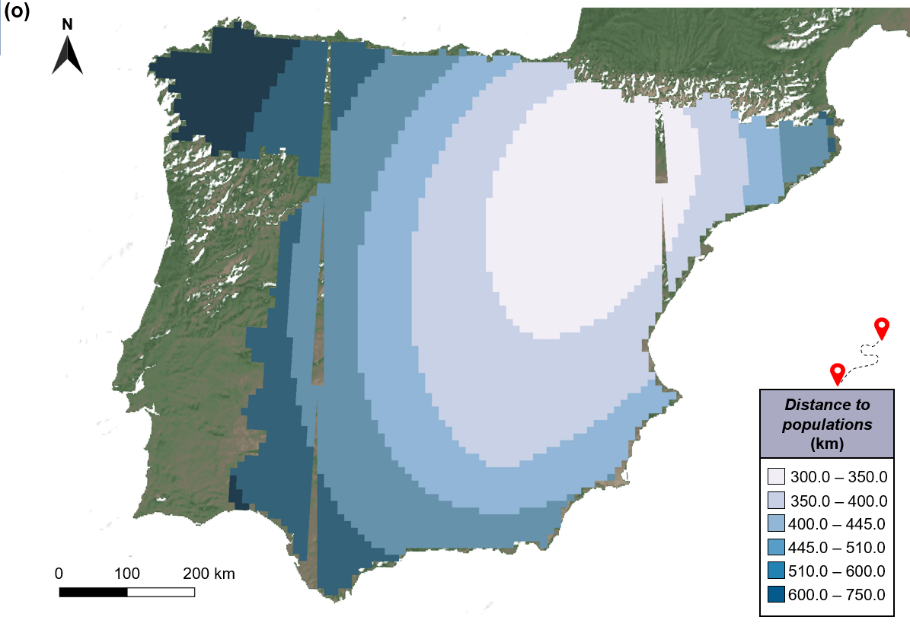


**Supplementary Fig. 2**

LRFs (≥ 350 km) of four breeding females from the Ebro valley population during the chick-rearing period (May-July). From above to below and from left to right the individual identity and year were T2W (2016), T2R (2018), T2L (2016), T2X (2016). The green area represents the main distribution area of the “dehesa” in the Iberian Peninsula (depicted from CORINE Land Cover). The black stars show the position of the nests. Maps were generated using QGIS 3.6.0 Noosa (https://www.qgis.org/es/site/)^90^.


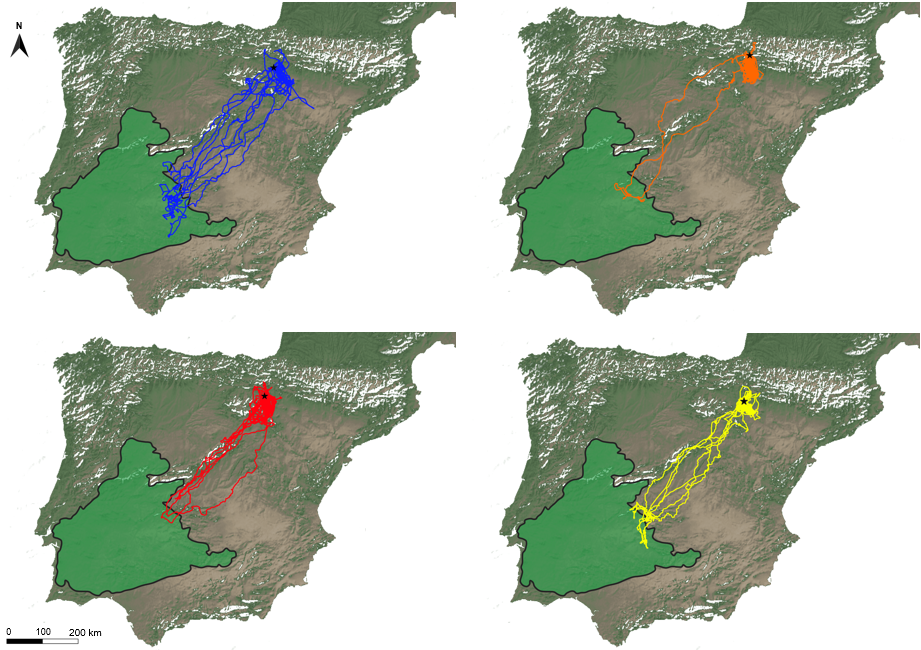


**Supplementary Table 1.**

Explanatory variables used to analyze the *Probability of presence* and *Number of populations* of GPS-tagged Eurasian griffon vultures in the UTM grid cells (10 x 10 km) covering mainland Spain.

| **Category** | **Name** | **Acronym** | **Description** |
| --- | --- | --- | --- |
| Physiography | *Slope* | *Slope* | Mean slope in degrees (a) |
| Vegetation | *Closed vegetation* | *CVeg* | Percentage of coverage classified as native forest (height>7m), reforestation (*Pinus spp.* And *Eucalyptus spp.*) and shrubland (b) |
|  | *Open vegetation* | *OVeg* | Percentage of coverage classified as scrubland (b) |
|  | *Dehesa* | *Deh* | Percentage of coverage classified as “dehesa” (b) |
| Urbanization | *Urban* | *Urb* | Urban surface in m^2^ (c) |
| Trophic | *Sheep/goats* | *Sheep* | Number of heads of sheep and goats in extensive and semi-extensive regime (d) |
|  | *Pigs* | *Pig* | Number of heads of pigs in extensive and semi-extensive regime (d) |
|  | *Cattle* | *Catt* | Number of heads of cattle in extensive and semi-extensive regime (d) |
|  | *Number livestock* | *NLiv* | Number of heads of livestock in extensive and semi-extensive regime (e)(+) |
|  | *Biomass livestock* | *Biom* | Amount of biomass (kg/year) of livestock. The weighted sum of the amount of biomass of livestock existing in all the municipalities included in the 50 x 50 km buffer (f)(++) |
|  | *Wild ungulates* | *WUng* | Sum of wild boars (*Sus scrofa*) and red deer (*Cervus elaphus*) hunted (g) |
| Competition | *Conspecifics* | *Consp* | Density of potentially foraging vultures (h) |
| Distance | *Distance to populations* | *DPop* | Euclidean distance between the centre of each grid-cell and the centroid of the studied population. |

1. Slope ASTER Global DEM spatial resolution 30 m^92, see 78^.
2. Based on the Forest Map of Spain 1:200,000^93^. We determined the percentage of coverage within each grid-cell of three vegetation categories: i) the sum of natural forest, reforested forest and shrub (Closed vegetation); ii) scrub and pasturelands (Open habitat); and iii) “dehesa”.
3. Numerical Cartographic Base 1:200,000 (BCN200 © National Geographic Institute of Spain)^94, see 78^.
4. Determined following Morales-Reyes *et al.* (2015)^37^. We obtained an abundance index for each grid-cell of sheep and goats, pigs and cattle in extensive and semi-extensive regimen (measured as total number of heads) based on municipality livestock censuses and the surface of each municipality in each cell.
5. Determined following Morales-Reyes *et al.* (2015)^37^. Sum of sheep/goats, pigs and cattle in extensive and semi-extensive regimen.
6. Determined following Morales-Reyes *et al.* (2015)^37^. Sum of sheep/goats, pigs and cattle in extensive and semi-extensive regimen.
7. Define an index of hunted wild ungulates as the sum of culled wild boars (*Sus scrofa*) and red deer (*Cervus elaphus*) (Ministerio de Agricultura, Pesca y Alimentación [MAPA], 2015-2017; Ministerio para la Transición Ecológica y el Reto Demográfico [MITECO], n.d.).
8. For each cell, we estimated an index of the number of breeding individuals in a circular buffer of 50 km radii, according to the census database of 2018^72^. We considered that most of the foraging activity of birds concentrates within 50 km from the breeding sites (Fig. 1 main text).

(+) Variable excluded of models because of its correlation (r>0.5) with Sheep/goats.

(++) Variable excluded of models because of its correlation (r>0.7) with Cattle.

**Supplementary Table 2.** We show the fraction (%) of days spent by GPS-tagged Eurasian griffon vultures of each population in locations >350 km far from the breeding area. The two seasons considered are split (S=summer; W=winter). For each population we show the number of birds by sex (F=females; M=males). Sample sizes in brackets below percentages.

|  | **% Days spent (>350km)** | | | | | | |
| --- | --- | --- | --- | --- | --- | --- | --- |
|  | **Female** | | | **Male** | | | **F\|M** |
| **Population\Season** | **S** | **W** | **S\|W** | **S** | **W** | **S\|W** | **Total** |
| Cádiz (10 M, 2 F) | 0  (216) | 0  (269) | 0  (485) | 6.96  (1,193) | 0  (1,320) | 3.30  (2,513) | 2.77  (2,998) |
| Catalonia (11 M, 2 F) | 0  (261) | 0  (122) | 0  (383) | 4.50  (1,379) | 0  (1,050) | 2.55  (2,429) | 2.20  (2,812) |
| Cazorla (19 M, 11 F) | 0.46  (5,825) | 0  (3,392) | 0.29  (9,217) | 3.16  (10,243) | 0.96  (6,490) | 2.31  (16,733) | 1.59  (25,950) |
| Ebro Valley (20 M, 16 F) | 23.47  (7,320) | 7.87  (4,347) | 17.66  (11,667) | 14.17  (6,915) | 20.06  (4,312) | 16.43  (11,227) | 17.06  (22,894) |
| French Pyrenees (13 M, 2 F) | 0  (194) | 0  (150) | 0  (344) | 6.66  (2,627) | 0  (1,361) | 4.39  (3,988) | 4.04  (4,332) |

**Supplementary Table 3.** Patterns of movements of GPS-tagged Eurasian griffon vultures of the Ebro valley population in relation to breeding performance. We show the number of birds of each sex that did LRFs (>350 km) and if these birds were breeding and in such a case, if the breeding attempt was successful.

| **Year** | **Sex** | **N total** | **N (>350 km)** | **% (>350 km)** | **N breed** | **N success** |
| --- | --- | --- | --- | --- | --- | --- |
| 2016 | M | 14 | 4 | 28.6 | 1 | 0 |
| 2016 | F | 15 | 8 | 53.3 | 6 | 5 |
| 2017 | M | 14 | 7 | 50.0 | 3 | 0 |
| 2017 | F | 12 | 9 | 75.0 | 6 | 1 |
| 2018 | M | 8 | 3 | 37.5 | 0 |  |
| 2018 | F | 8 | 5 | 62.5 | 3 | 1 |

**Supplementary Table 4.**

Factors affecting the probability that a Eurasian griffon vulture daily location be at more than 350 km from the individual’s nest or activity center. We show the ten top-ranked competing models resulting of the GLMM procedure (plus the null model).

| **Model** | **AICc** | **∆AICc** | **AICcWt** | **Cumulative Weight** |
| --- | --- | --- | --- | --- |
| ***Population* + *Season* + *Sex* + *Season*Sex*** | **5168.984** | **0** | **0.813** | **0.813** |
| *Population* + *Season* + *Sex* + *Population*Sex* + *Season*Sex* | 5172.633 | 3.65 | 0.131 | 0.944 |
| *Season* + *Sex* + *Season*Sex* | 5174.323 | 5.34 | 0.056 | 1.000 |
| *Population* + *Season* | 5427.962 | 258.98 | 0.000 | 1.000 |
| *Population* + *Season* + *Sex* | 5429.725 | 260.74 | 0.000 | 1.000 |
| *Population* + *Season* + *Sex* + *Population*Sex* | 5433.367 | 264.38 | 0.000 | 1.000 |
| *Season* + *Sex* | 5435.411 | 266.43 | 0.000 | 1.000 |
| *Season* | 5436.231 | 267.25 | 0.000 | 1.000 |
| *Population* | 5730.738 | 561.75 | 0.000 | 1.000 |
| *Population* + *Sex* | 5732.496 | 563.51 | 0.000 | 1.000 |
| Null | 5738.660 | 569.68 | 0.000 | 1.000 |

**Supplementary Table 5.**

Description of the final model evaluating the probability that a daily location is more than 350 km from the individual’s nest or activity center. We show the estimates, standard errors (SE) and 95% confidence intervals (CI lower and CI upper) of the explanatory variable. In bold informative parameters. Marginal (R^2^m) and conditional (R^2^c) coefficients of determination are also shown^89^.

| **Variables** | **Estimate** | **SE** | **CI lower** | **CI upper** |
| --- | --- | --- | --- | --- |
| **Intercept** | **-8.934** | **2.187** | **-13.220** | **-4.659** |
| *Population (Catalonia)* | -0.131 | 2.707 | -5.436 | 5.175 |
| *Population (Cazorla)* | -0.116 | 2.139 | -4.308 | 4.077 |
| *Population (Ebro valley)* | 2.980 | 2.103 | -1.143 | 7.102 |
| *Population (French Pyrenees)* | 0.354 | 2.554 | -4.652 | 5.360 |
| ***Season (Summer)*** | **1.450** | **0.067** | **1.319** | **1.581** |
| *Sex (Male)* | 0.581 | 1.148 | -1.668 | 2.831 |
| ***Season (Summer) : Sex (Male)*** | **-1.384** | **0.088** | **-1.555** | **-1.212** |
| R^2^m | 0.097 |  |  |  |
| R^2^c | 0.999 |  |  |  |

**Supplementary Table 6.**

Effects of individual’s *Sex* and *Breeding success* on the probability that a daily location is more than 350 km away of the nest or activity center in vultures from the Ebro valley population. We show all competing models resulting of the GLM procedure. Only one model (bolded) was within ∆AICc<2.

| **Model** | **AICc** | **∆AICc** | **AICcWt** | **Cumulative weight** |
| --- | --- | --- | --- | --- |
| ***Breeding success* + *Sex*** | **86.8** | **0.00** | **0.855** | **0.855** |
| *Breeding success* | 90.6 | 3.81 | 0.127 | 0.982 |
| *Sex* | 95.5 | 8.72 | 0.011 | 0.993 |
| Null | 96.5 | 9.77 | 0.006 | 1.000 |

**Supplementary Table 7.**

Result of the best model of the probability that a daily location is far than 350 km of the individual’s nest or activity center within the Ebro valley population. We show the estimates, standard errors (SE) and 95% confidence intervals (CI lower and CI upper) of the explanatory variables; all the parameters were informative. The coefficient of determination (R^2^) is also shown^89^.

|  | **Estimate** | **SE** | **CI lower** | **CI upper** |
| --- | --- | --- | --- | --- |
| (Intercept) | 1.572 | 0.511 | 0.652 | 2.695 |
| *Sex* *(Male)* | -1.3661 | 0.590 | -2.613 | -0.263 |
| *Breeding success (Yes)* | -1.9234 | 0.631 | -3.260 | -0.753 |
| R^2^=0.185 | | |  |  |
|  | | |  |  |

**Supplementary Table 8.**

Factors promoting the *Probability of presence* of GPS-tagged Eurasian griffon vultures at a 10x10 km UTM grid cell in mainland Spain. We show the ten top-ranked competing models (plus the null model). Alternative models (∆AICc<2) are bolded.

| **Model** | **AICc** | **∆AICc** | **AICcWt** | **Cumulative Weight** |
| --- | --- | --- | --- | --- |
| ***WUng + Sheep + Catt + Deh + Consp + DPop + OVeg + Pig + Slope*** | **4505.80** | **0** | **0.333** | **0.333** |
| ***WUng + Sheep + Catt + Deh + Consp + DPop + Urb + OVeg + Pig + Slope*** | **4506.27** | **0.46** | **0.264** | **0.597** |
| ***WUng + Sheep + Catt + CVeg + Deh + Consp + DPop + OVeg + Pig + Slope*** | **4507.21** | **1.41** | **0.165** | **0.762** |
| ***WUng + Sheep + Catt + CVeg + Deh + Consp + DPop + Urb + OVeg + Pig + Slope*** | **4507.73** | **1.93** | **0.127** | **0.889** |
| *WUng + Sheep + Cattle + Deh + Consp + DPop + OVeg + Pig* | 4510.43 | 4.62 | 0.033 | 0.922 |
| *WUng + Sheep + Catt + Deh + Consp + DPop + Urb + OVeg + Pig* | 4510.69 | 4.89 | 0.029 | 0.951 |
| *WUng + Sheep + Catt + CVeg + Deh + Consp + DPop + OVeg + Pig* | 4511.33 | 5.52 | 0.021 | 0.972 |
| *WUng + Sheep + Catt + CVeg + Deh + Consp + DPop + Urb + OVeg + Pig* | 4511.59 | 5.78 | 0.018 | 0.990 |
| *WUng + Sheep + Catt + Deh + Consp + DPop + Pig + Slope* | 4515.54 | 9.73 | 0.003 | 0.993 |
| *WUng + Sheep + Catt + Deh + Consp + DPop + Urb + Pig + Slope* | 4515.55 | 9.75 | 0.003 | 0.996 |
| Null | 6147.26 | 1641.45 | 0.000 | 1.190 |

**Supplementary Table 9.**

Averaged GLM model, and Alternative GLM models (∆AICc<2) **(**1 to 4, order corresponds to the one shown bolded in Supplementary Table 8), of the *Probability of* *presence* of GPS-tagged Eurasian griffon vultures in the 10x10 km UTM grid cell from mainland Spain. Estimate, standard errors (SE) and 95% confidence intervals (CI lower and CI upper) are shown. Variables receiving the strongest support (i.e., estimates do not overlap zero) are bolded. The coefficient of determination (R^2^) is also shown^89^.

| **Averaged Model** | | | | |
| --- | --- | --- | --- | --- |
| *WUng + Sheep + Catt + CVeg + Deh + Consp + DPop + Urb + OVeg + Pig + Slope* | | | | |
| **Variable** | **Estimate** | **SE** | **CI lower** | **CI upper** |
| **Intercept** | **-0.305** | **0.041** | **-0.386** | **-0.225** |
| ***Wild ungulates*** | **0.712** | **0.043** | **0.628** | **0.797** |
| ***Sheep/goats*** | **0.916** | **0.069** | **0.780** | **1.052** |
| ***Cattle*** | **0.256** | **0.065** | **0.127** | **0.384** |
| ***Dehesa*** | **0.388** | **0.057** | **0.276** | **0.501** |
| ***Conspecifics*** | **0.215** | **0.038** | **0.140** | **0.290** |
| ***Distance to populations*** | **-0.604** | **0.057** | **-0.716** | **-0.493** |
| ***Open vegetation*** | **0.129** | **0.038** | **0.055** | **0.203** |
| ***Pigs*** | **0.693** | **0.112** | **0.474** | **0.912** |
| ***Slope*** | **0.109** | **0.045** | **0.020** | **0.197** |
| *Urban* | -0.020 | 0.033 | -0.118 | 0.028 |
| *Closed vegetation* | -0.013 | 0.036 | -0.146 | 0.065 |

| **Model 1** |  | | | | |
| --- | --- | --- | --- | --- | --- |
| *WUng + Sheep + Catt + Deh + Consp + DPop + OVeg + Pig + Slope* | | | | | |
| **Variable** | | **Estimate** | **SE** | **CI lower** | **CI upper** |
| **Intercept** | | **-0.305** | **0.041** | **-0.385** | **-0.224** |
| ***Wild ungulates*** | | **0.714** | **0.043** | **0.630** | **0.799** |
| ***Sheep/goats*** | | **0.921** | **0.069** | **0.788** | **1.059** |
| ***Cattle*** | | **0.255** | **0.065** | **0.126** | **0.383** |
| ***Dehesa*** | | **0.391** | **0.057** | **0.281** | **0.505** |
| ***Conspecifics*** | | **0.214** | **0.038** | **0.140** | **0.288** |
| ***Distance to populations*** | | **-0.605** | **0.057** | **-0.717** | **-0.495** |
| ***Open vegetation*** | | **0.129** | **0.038** | **0.055** | **0.203** |
| ***Pigs*** | | **0.695** | **0.111** | **0.486** | **0.914** |
| ***Slope*** | | **0.101** | **0.039** | **0.024** | **0.178** |
| R^2^ | | 0.305 |  |  |  |
| Null Deviance (d.f.) | | 6143.3 | (4546) |  |  |
| Residual Deviance (d.f.) | | 4485.8 | (4537) |  |  |

| **Model 2** |  | | | | |
| --- | --- | --- | --- | --- | --- |
| *WUng + Sheep + Catt + Deh + Consp + DPop + Urb + OVeg + Pig + Slope* | | | | | |
| **Variable** | | **Estimate** | **SE** | **CI lower** | **CI upper** |
| **Intercept** | | **-0.306** | **0.041** | **-0.386** | **-0.225** |
| ***Wild ungulates*** | | **0.710** | **0.043** | **0.626** | **0.795** |
| ***Sheep/goats*** | | **0.911** | **0.069** | **0.777** | **1.050** |
| ***Cattle*** | | **0.259** | **0.065** | **0.130** | **0.387** |
| ***Dehesa*** | | **0.387** | **0.057** | **0.277** | **0.501** |
| ***Conspecifics*** | | **0.213** | **0.038** | **0.138** | **0.287** |
| ***Distance to populations*** | | **-0.608** | **0.057** | **-0.719** | **-0.497** |
| ***Open vegetation*** | | **0.127** | **0.038** | **0.053** | **0.201** |
| ***Pigs*** | | **0.687** | **0.111** | **0.477** | **0.915** |
| ***Slope*** | | **0.100** | **0.039** | **0.023** | **0.176** |
| *Urban* | | -0.046 | 0.037 | -0.121 | 0.256 |
| R^2^ | | 0.306 |  |  |  |
| Null Deviance (d.f.) | | 6143.3 | (4546) |  |  |
| Residual Deviance (d.f.) | | 4484.2 | (4536) |  |  |

| **Model 3** |  | | | | |
| --- | --- | --- | --- | --- | --- |
| *WUng + Sheep + Catt + CVeg + Deh + Consp + DPop + OVeg + Pig + Slope* | | | | | |
| **Variable** | | **Estimate** | **SE** | **CI lower** | **CI upper** |
| **Intercept** | | **-0.305** | **0.041** | **-0.386** | **-0.224** |
| ***Wild ungulates*** | | **0.715** | **0.043** | **0.631** | **0.800** |
| ***Sheep/goats*** | | **0.919** | **0.069** | **0.787** | **1.058** |
| ***Cattle*** | | **0.252** | **0.065** | **0.124** | **0.381** |
| ***Dehesa*** | | **0.389** | **0.057** | **0.278** | **0.503** |
| ***Conspecifics*** | | **0.220** | **0.039** | **0.144** | **0.296** |
| ***Distance to populations*** | | **-0.601** | **0.057** | **-0.714** | **-0.490** |
| ***Open vegetation*** | | **0.132** | **0.038** | **0.058** | **0.206** |
| ***Pigs*** | | **0.700** | **0.112** | **0.490** | **0.929** |
| ***Slope*** | | **0.127** | **0.051** | **0.026** | **0.227** |
| *Closed vegetation* | | -0.041 | 0.054 | -0.147 | 0.063 |
| R^2^ | | 0.306 |  |  |  |
| Null Deviance (d.f.) | | 6143.3 | (4546) |  |  |
| Residual Deviance (d.f.) | | 4485.2 | (4536) |  |  |

| **Model 4** |  | | | | |  |
| --- | --- | --- | --- | --- | --- | --- |
| *WUng + Sheep + Catt + CVeg + Deh + Consp + DPop + Urb + OVeg + Pig + Slope* | | | | | | |
| **Variable** | | **Estimate** | **SE** | **CI lower** | **CI upper** |  |
| **Intercept** | | **-0.306** | **0.041** | **-0.386** | **-0.225** |  |
| ***Wild ungulates*** | | **0.710** | **0.043** | **0.626** | **0.796** |  |
| ***Sheep/goats*** | | **0.909** | **0.069** | **0.776** | **1.048** |  |
| ***Cattle*** | | **0.256** | **0.065** | **0.128** | **0.384** |  |
| ***Dehesa*** | | **0.385** | **0.057** | **0.274** | **0.500** |  |
| ***Conspecifics*** | | **0.218** | **0.039** | **0.142** | **0.294** |  |
| ***Distance to populations*** | | **-0.603** | **0.057** | **-0.715** | **-0.492** |  |
| ***Open vegetation*** | | **0.130** | **0.038** | **0.055** | **0.204** |  |
| ***Pigs*** | | **0.691** | **0.112** | **0.481** | **0.920** |  |
| ***Slope*** | | **0.124** | **0.051** | **0.024** | **0.224** |  |
| *Urban* | | -0.045 | 0.037 | -0.120 | 0.026 |  |
| *Closed vegetation* | | -0.040 | 0.054 | -0.145 | 0.065 |  |
| R^2^ | | 0.306 |  |  |  |  |
| Null Deviance (d.f.) | | 6143.3 | (4546) |  |  |  |
| Residual Deviance (d.f.) | | 4483.7 | (4535) |  |  |  |

**Supplementary Table 10.**

Factors affecting the *Number of populations* of GPS-tagged Eurasian griffon vultures sharing 10x10 km UTM grid cells in mainland Spain. We show the ten top-ranked competing models (plus the null model). Alternative models (∆AICc<2) are bolded.

| **Model** | **AICc** | **∆AICc** | **AICcWt** | **Cumulative Weight** |
| --- | --- | --- | --- | --- |
| ***WUng + Sheep + Catt + Deh + Consp + DPop + Urb + OVeg + Pig*** | **7234.03** | **0.000** | **0.389** | **0.389** |
| ***WUng + Sheep + Catt + CVeg + Deh + Consp + DPop + Urb + OVeg + Pig*** | **7234.77** | **0.740** | **0.268** | **0.657** |
| ***WUng + Sheep + Catt + Deh + Consp + DPop + Urb + OVeg + Pig + Slope*** | **7235.24** | **1.203** | **0.213** | **0.870** |
| *WUng + Sheep + Catt + CVeg + Deh + Consp + DPop + Urb + OVeg + Pig + Slope* | 7236.72 | 2.684 | 0.101 | 0.971 |
| *WUng + Sheep + Catt + Deh + Consp + DPop + Urb + Pig* | 7241.92 | 7.886 | 0.008 | 0.979 |
| *WUng + Sheep + Catt + CVeg + Deh + Consp + DPop + Urb + Pig* | 7242.47 | 8.432 | 0.006 | 0.985 |
| *WUng + Sheep + Catt + Deh + Consp + DPop + Urb + Pig + Slope* | 7242.80 | 8.763 | 0.005 | 0.990 |
| *WUng + Sheep + Catt + CVeg + Deh + Consp + DPop + Urb + Pig + Slope* | 7243.58 | 9.545 | 0.003 | 0.993 |
| *WUng + Sheep + Catt + Deh + Consp + Urb + OVeg + Pig* | 7244.18 | 10.146 | 0.002 | 0.995 |
| *WUng + Sheep + Catt + Deh + Consp + DPop + OVeg + Pig* | 7244.78 | 10.749 | 0.002 | 0.997 |
| Null | 8888.11 | 1654.076 | 0.000 | 0.997 |

**Supplementary Table 11.**

Averaged GLM model, and Alternative GLM models (∆AICc<2) **(**1 to 3, order corresponds to the one shown bolded in Supplementary Table 10), of the *Number of populations* of GPS-tagged Eurasian griffon vultures sharing 10x10 km UTM grid cells from mainland Spain. Estimate, standard errors (SE) and 95% confidence intervals (CI lower and CI upper) of the variables. Variables receiving the strongest support (i.e., estimates do not overlap zero) are bolded. The coefficient of determination (R^2^) is also shown^89^.

| **Averaged model** |  |  |  |  |  |
| --- | --- | --- | --- | --- | --- |
| *WUng + Sheep + Catt + CVeg + Deh + Consp + DPop + Urb + OVeg + Pig + Slope* | | | | | |
| **Variable** | **Estimate** | **SE** | **CI lower** | **CI upper** |  |
| **Intercept** | **-2.364** | **0.026** | **-2.414** | **-2.314** |  |
| ***Wild ungulates*** | **0.391** | **0.021** | **0.349** | **0.433** |  |
| ***Sheep/goats*** | **0.354** | **0.020** | **0.314** | **0.394** |  |
| ***Cattle*** | **-0.124** | **0.027** | **-0.177** | **-0.071** |  |
| ***Dehesa*** | **0.178** | **0.019** | **0.140** | **0.217** |  |
| ***Conspecifics*** | **0.177** | **0.024** | **0.129** | **0.225** |  |
| ***Distance to populations*** | **-0.132** | **0.032** | **-0.194** | **-0.069** |  |
| ***Open vegetation*** | **0.069** | **0.022** | **0.026** | **0.112** |  |
| ***Pigs*** | **0.193** | **0.021** | **0.152** | **0.234** |  |
| *Slope* | 0.006 | 0.016 | -0.028 | 0.075 |  |
| ***Urban*** | **-0.105** | **0.032** | **-0.168** | **-0.041** |  |
| *Closed vegetation* | 0.009 | 0.021 | -0.023 | 0.084 |  |

| **Model 1** |  | | | | |
| --- | --- | --- | --- | --- | --- |
| *WUng + Sheep + Catt + Deh + Consp + DPop + Urb + OVeg + Pig* | | | | | |
| **Variable** | | **Estimate** | **SE** | **CI lower** | **CI upper** |
| **Intercept** | | **-2.364** | **0.026** | **-2.415** | **-2.314** |
| ***Wild ungulates*** | | **0.392** | **0.021** | **0.350** | **0.433** |
| ***Sheep/goats*** | | **0.353** | **0.020** | **0.313** | **0.393** |
| ***Cattle*** | | **-0.125** | **0.027** | **-0.178** | **-0.072** |
| ***Dehesa*** | | **0.177** | **0.019** | **0.139** | **0.214** |
| ***Conspecifics*** | | **0.180** | **0.024** | **0.134** | **0.226** |
| ***Distance to populations*** | | **-0.128** | **0.031** | **-0.190** | **-0.067** |
| ***Open vegetation*** | | **0.071** | **0.021** | **0.029** | **0.113** |
| ***Pigs*** | | **0.193** | **0.021** | **0.152** | **0.234** |
| ***Urban*** | | **-0.105** | **0.032** | **-0.171** | **-0.044** |
| R^2^ | | 0.308 |  |  |  |
| Null Deviance (d.f.) | | 5423.0 | (4546) |  |  |
| Residual Deviance (d.f.) | | 3750.9 | (4537) |  |  |

| **Model 2** |  | | | | |
| --- | --- | --- | --- | --- | --- |
| *WUng + Sheep + Catt + CVeg + Deh + Consp + DPop + Urb + OVeg + Pig* | | | | | |
| **Variable** | | **Estimate** | **SE** | **CI lower** | **CI upper** |
| **Intercept** | | **-2.364** | **0.026** | **-2.415** | **-2.314** |
| ***Wild ungulates*** | | **0.390** | **0.021** | **0.349** | **0.432** |
| ***Sheep/goats*** | | **0.355** | **0.020** | **0.315** | **0.395** |
| ***Cattle*** | | **-0.123** | **0.027** | **-0.177** | **-0.070** |
| ***Dehesa*** | | **0.180** | **0.020** | **0.142** | **0.218** |
| ***Conspecifics*** | | **0.172** | **0.025** | **0.123** | **0.220** |
| ***Distance to populations*** | | **-0.137** | **0.032** | **-0.200** | **-0.074** |
| ***Open vegetation*** | | **0.067** | **0.022** | **0.024** | **0.109** |
| ***Pigs*** | | **0.194** | **0.021** | **0.153** | **0.235** |
| ***Urban*** | | **-0.105** | **0.032** | **-0.170** | **-0.044** |
| ***Closed vegetation*** | | **0.031** | **0.027** | **-0.023** | **0.084** |
| R^2^ | | 0.308 |  |  |  |
| Null Deviance (d.f.) | | 5423.0 | (4546) |  |  |
| Residual Deviance (d.f.) | | 3749.6 | (4536) |  |  |

| **Model 3** |  | | | | |
| --- | --- | --- | --- | --- | --- |
| *WUng + Sheep + Catt + Deh + Consp + DPop + Urb + OVeg + Pig + Slope* | | | | | |
| **Variable** | | **Estimate** | **SE** | **CI lower** | **CI upper** |
| **Intercept** | | **-2.364** | **0.026** | **-2.414** | **-2.314** |
| ***Wild ungulates*** | | **0.390** | **0.021** | **0.349** | **0.432** |
| ***Sheep/goats*** | | **0.354** | **0.020** | **0.315** | **0.394** |
| ***Cattle*** | | **-0.124** | **0.027** | **-0.178** | **-0.072** |
| ***Dehesa*** | | **0.179** | **0.020** | **0.141** | **0.217** |
| ***Conspecifics*** | | **0.176** | **0.024** | **0.128** | **0.223** |
| ***Distance to populations*** | | **-0.132** | **0.032** | **-0.195** | **-0.071** |
| ***Open vegetation*** | | **0.067** | **0.022** | **0.024** | **0.110** |
| ***Pigs*** | | **0.193** | **0.021** | **0.152** | **0.234** |
| ***Slope*** | | **0.024** | **0.026** | **-0.028** | **0.074** |
| ***Urban*** | | **-0.105** | **0.032** | **-0.171** | **-0.044** |
| R^2^ | | 0.308 |  |  |  |
| Null Deviance (d.f.) | | 5423.0 | (4546) |  |  |
| Residual Deviance (d.f.) | | 3750.1 | (4536) |  |  |
